# Liver perfusion MRI in a rodent model of cirrhosis: Agreement with bulk‐flow phase‐contrast MRI and noninvasive evaluation of inflammation in chronic liver disease using flow‐sensitive alternating inversion recovery arterial spin labelling and tissue T1

**DOI:** 10.1002/nbm.4423

**Published:** 2020-10-07

**Authors:** Manil Dinesh Chouhan, Rajiv Ramasawmy, Alan Bainbridge, Adrienne Campbell‐Washburn, Steve Halligan, Nathan Davies, Simon Walker‐Samuel, Mark F. Lythgoe, Rajeshwar Prosad Mookerjee, Stuart Andrew Taylor

**Affiliations:** ^1^ UCL Centre for Medical Imaging London UK

**Keywords:** arterial spin labelling, chronic liver disease, cirrhosis, inflammation, liver perfusion, liver T1, phase‐contrast MRI, sepsis

## Abstract

Noninvasive measurements of liver perfusion and fibrosis in cirrhotic small animals can help develop treatments for haemodynamic complications of liver disease. Here, we measure liver perfusion in cirrhotic rodents using flow‐sensitive alternating inversion recovery arterial spin labelling (FAIR ASL), evaluating agreement with previously validated caval subtraction phase‐contrast magnetic resonance imaging (PCMRI) total liver blood flow (TLBF). Baseline differences in cirrhotic rodents and the haemodynamic effects of acute inflammation were investigated using FAIR ASL and tissue T1. Sprague–Dawley rats (nine bile duct ligated [BDL] and ten sham surgery controls) underwent baseline hepatic FAIR ASL with T1 measurement and caval subtraction PCMRI (with two‐dimensional infra‐/supra‐hepatic inferior vena caval studies), induction of inflammation with intravenous lipopolysaccharide (LPS) and repeat liver FAIR ASL with T1 measurement after ~90 minutes. The mean difference between FAIR ASL hepatic perfusion and caval subtraction PCMRI TLBF was −51 ± 30 ml/min/100 g (Bland–Altman 95% limits‐of‐agreement ±258 ml/min/100 g). The FAIR ASL coefficient of variation was smaller than for caval subtraction PCMRI (29.3% vs 50.1%; *P* = .03). At baseline, FAIR ASL liver perfusion was lower in BDL rats (199 ± 32 ml/min/100 g vs sham 316 ± 24 ml/min/100 g; *P* = .01) but liver T1 was higher (BDL 1533 ± 50 vs sham 1256 ± 18 ms; *P* = .0004). Post‐LPS FAIR ASL liver perfusion response differences were observed between sham/BDL rats (*P* = .02), approaching significance in sham (+78 ± 33 ml/min/100 g; *P* = .06) but not BDL rats (−49 ± 40 ml/min/100 g; *P* = .47). Post‐LPS differences in liver tissue T1 were nonsignificant (*P* = .35). FAIR ASL hepatic perfusion and caval subtraction PCMRI TLBF agreement was modest, with significant baseline FAIR ASL liver perfusion and tissue T1 differences in rodents with advanced cirrhosis compared with controls. Following inflammatory stress, differences in hepatic perfusion response were detected between cirrhotic/control animals, but liver T1 was unaffected. Findings underline the potential of FAIR ASL in the assessment of vasoactive treatments for patients with chronic liver disease and inflammation.

## Graphical abstract

Flow‐sensitive alternating inversion recovery arterial spin labelling (FAIR ASL) was used to measure liver perfusion and T1 at baseline and following inflammatory stress in cirrhotic and control rats. Baseline agreement of FAIR ASL liver perfusion with previously validated caval subtraction phase‐contrast MRI was modest. Following inflammatory stress, significant differences in hepatic perfusion response were detected between cirrhotic and control animals but liver T1 was unaffected, highlighting the potential of FAIR ASL in the assessment of chronic liver disease and inflammation.

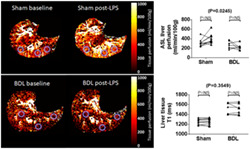

**Abbreviations used**
ACLF: acute‐on‐chronic liver failure
ANOVA: analysis of variance
ASL: arterial spin labelling
BDL: bile duct ligated
CoV: coefficient of variation
CT: computerised tomography
DCE: dynamic contrast enhanced
FAIR: flow‐sensitive alternating inversion recovery
HA: hepatic artery
ICG: indocyanine green
LoA: limits‐of‐agreement
LPS: lipopolysaccharide
MRI: magnetic resonance imaging
NASH: nonalcoholic steatohepatitis
PCMRI: phase‐contrast magnetic resonance imaging
PV: portal vein
ROI: region of interest
SNR: signal‐to‐noise ratio
TE: echo time
TI: inversion time
TLBF: total liver blood flow
TR: repetition time
V_enc_
: velocity‐encoding setting

## 1 INTRODUCTION

Liver cirrhosis causes profound changes in hepatic blood flow, modifying contributions from the portal vein (PV) and hepatic artery (HA), total liver blood flow (TLBF) and downstream tissue perfusion.^1^ This reflects complex vascular changes in portal hypertension and could potentially assess the impact of novel vasoactive drugs to treat complications of liver disease such as portal hypertension. However, noninvasive quantification is challenging and haemodynamic changes in the setting of acute inflammation super‐imposed on chronic liver disease are even less well understood. Acute‐on‐chronic liver failure (ACLF), a recently defined syndrome, is often precipitated by sepsis and is characterised by severe inflammatory responses, deranged hepatic function, extrahepatic organ failure and high mortality.^2^, ^3^ Hepatic and systemic circulatory haemodynamics are perturbed significantly following inflammation,^4^ and while therapeutic haemodynamic modulatory agents may improve clinical outcomes, response measurement is difficult.^5^

Small animal models of liver disease are useful, but evaluating hepatic perfusion is challenging because reference standards are invasive (and may themselves confound measurement), or are unfeasible in disease.^6^ Cross‐sectional imaging such as dynamic contrast enhanced (DCE) CT/MRI may overcome some of these limitations but it requires additional vascular access and relies on exogenous contrast agents, which may also confound measurement. Short interval repeat studies to monitor dynamic vascular changes are also difficult.^1^

Arterial spin labelling (ASL) is an MRI method that employs endogenous blood water as a tracer: blood is labelled by inversion and exchange of inverted blood with tissue magnetisation is used to generate perfusion‐sensitised images. Signal difference between labelled perfusion‐weighted and static tissue control images is used to measure tissue perfusion.^7^ Flow‐sensitive alternating inversion recovery (FAIR) is a pulsed ASL method that relies on the difference between T1 measurements following slice‐selective and global inversions, where acceleration of T1 recovery after the slice‐selective inversion caused by noninverted spins perfusing the imaged slice is perfusion‐weighted. It has been used previously to demonstrate differences in compensated vs decompensated chronic liver disease patients^8^ and changes in liver tumour perfusion in mice.^9^ Static liver tissue T1 maps generated by FAIR ASL are also potentially useful, as liver T1 is known to vary with fibrosis severity^10^ and has also been proposed as a measure of tissue inflammation.^11^ Histological endotoxin‐induced acute hepatic inflammation has been demonstrated previously,^12^, ^13^ moreover noninvasive measurement of liver inflammation would be clinically useful, particularly in the context of chronic inflammatory conditions such as nonalcoholic steatohepatitis (NASH).^14^

Caval subtraction phase‐contrast MRI (PCMRI) is a noninvasive method to estimate bulk TLBF that has been previously invasively validated in cirrhotic rodent models.^15^ Although bulk vessel flow and tissue perfusion are different haemodynamic parameters, they are inherently related. Because invasive validation is particularly challenging in small animals, demonstrating good agreement between these methods would increase confidence that FAIR ASL measures tissue perfusion accurately.

In this study we used a rodent model of cirrhosis to assess agreement between FAIR ASL measurements of hepatic perfusion and caval subtraction PCMRI, to characterise baseline differences between cirrhotic and control animals, and to investigate the effects of super‐imposed inflammatory stress on FAIR ASL liver perfusion and tissue T1 in the setting of chronic liver disease.

## 2 MATERIALS AND METHODS

### 2.1 Animal preparation

All experiments were conducted in accordance with UK Home Office guidelines from the Animals in Scientific Procedures Act (1986), with approval from the Animal Care Ethical Committee of University College London. Experiments were performed on healthy male Sprague–Dawley rats (Charles River, Margate, UK, weight 250‐300 g, age 8 weeks old) with normal liver function.

Nineteen healthy animals were subjected to either sham surgery (laparotomy without bile duct ligation, *n* = 10) or bile duct ligation (*n* = 9).^16^ Animals were maintained for 4‐5 weeks for the development of cirrhosis in the bile duct ligated (BDL) cohort. After induction with isoflurane (2% in 1 L O_2_/min), a fine bore polyethylene line (Portex, Smiths Medical, Kent, UK) was inserted into the jugular vein, just prior to scanning. Once transferred to the scanner, anaesthesia was maintained at a constant level using isoflurane (~2% in 1 L O_2_/min). Temperature was monitored using a rectal probe (SA Instruments, New York, NY, USA) and maintained at 36‐38°C using warm air and pipes with circulating warm water. Respiratory bellows (SA Instruments) were placed over the upper abdomen and cardiac monitoring was undertaken using a triple‐electrode single‐lead system (SA Instruments). All procedures were performed by the study coordinator (MDC, a radiology research fellow qualified in animal handling).

### 2.2 Study design and inflammatory stress studies

**FIGURE 1:**
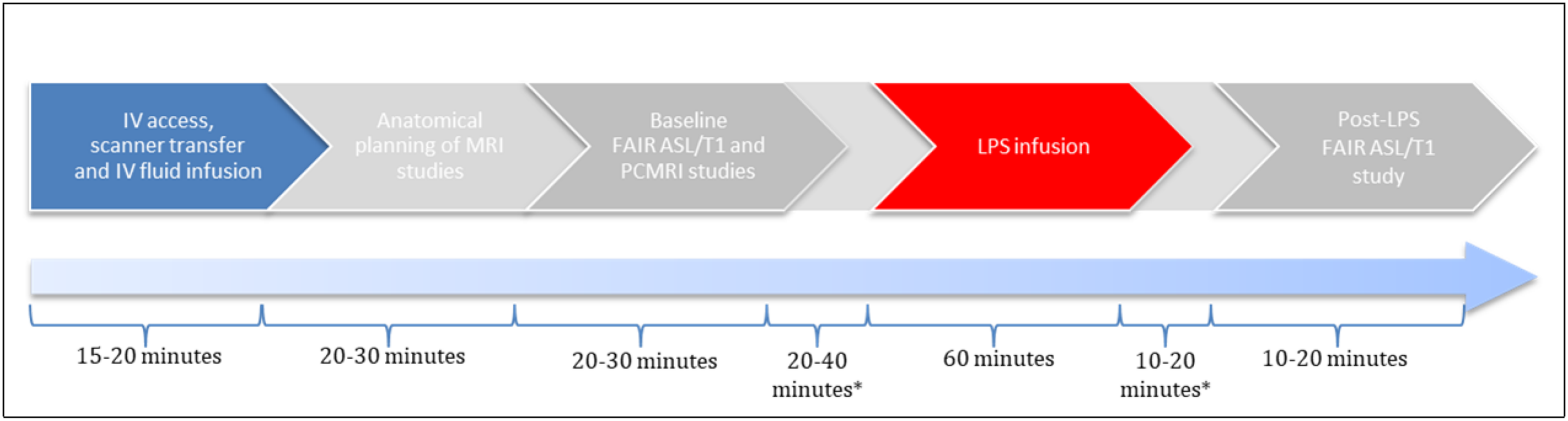
Experimental protocol, with approximate timings of each phase (*intervals during which other imaging data were collected, not presented in this study)

The jugular venous line was used to infuse intravenous normal saline continuously at a rate of 8 ml/kg/hour for the duration of the experiment (~1.5‐2.5 hours). After baseline MRI, inflammation was induced with a 60‐minute infusion of 0.3 mg/kg lipopolysaccharide (*Escherichia coli* LPS, Sigma Aldrich, UK). Normal saline fluid resuscitation infusion was then resumed and post‐LPS FAIR ASL/liver tissue T1 studies were undertaken after 10‐20 minutes (Figure 1).

### 2.3 Scanning and image analysis protocol

Scans were performed on a 9.4 T MRI unit (Agilent Technologies, Oxford, UK) using the sequence parameters listed in Table 1. Axial and coronal respiratory‐gated gradient‐echo anatomical imaging was used for planning.

**TABLE 1:** Sequence parameters

	Anatomical images (gradient‐echo sequence)	FAIR ASL/T1 mapping	PCMRI
TR/TE (milliseconds)	8.2/5.6	2.3/1.18	10/1.2
Flip angle (°)	20	8	10
Matrix size (pixels)	128 x 128	128 x 128	192 x 192
Field of view (mm)	80 x 80	60 x 60	40 x 40
Spatial resolution (mm^2^)	0.625 x 0.625	0.469 x 0.469	0.208 x 0.208
Slice thickness (mm)	2	2	2
Slice gap (mm)	4.5	‐	‐
Inversion time spacing (ms)	‐	110	‐
Inversion readouts	‐	50	‐
Cardiac cycle phases	‐	‐	12‐15

### 2.4 FAIR ASL perfusion

**FIGURE 2:**
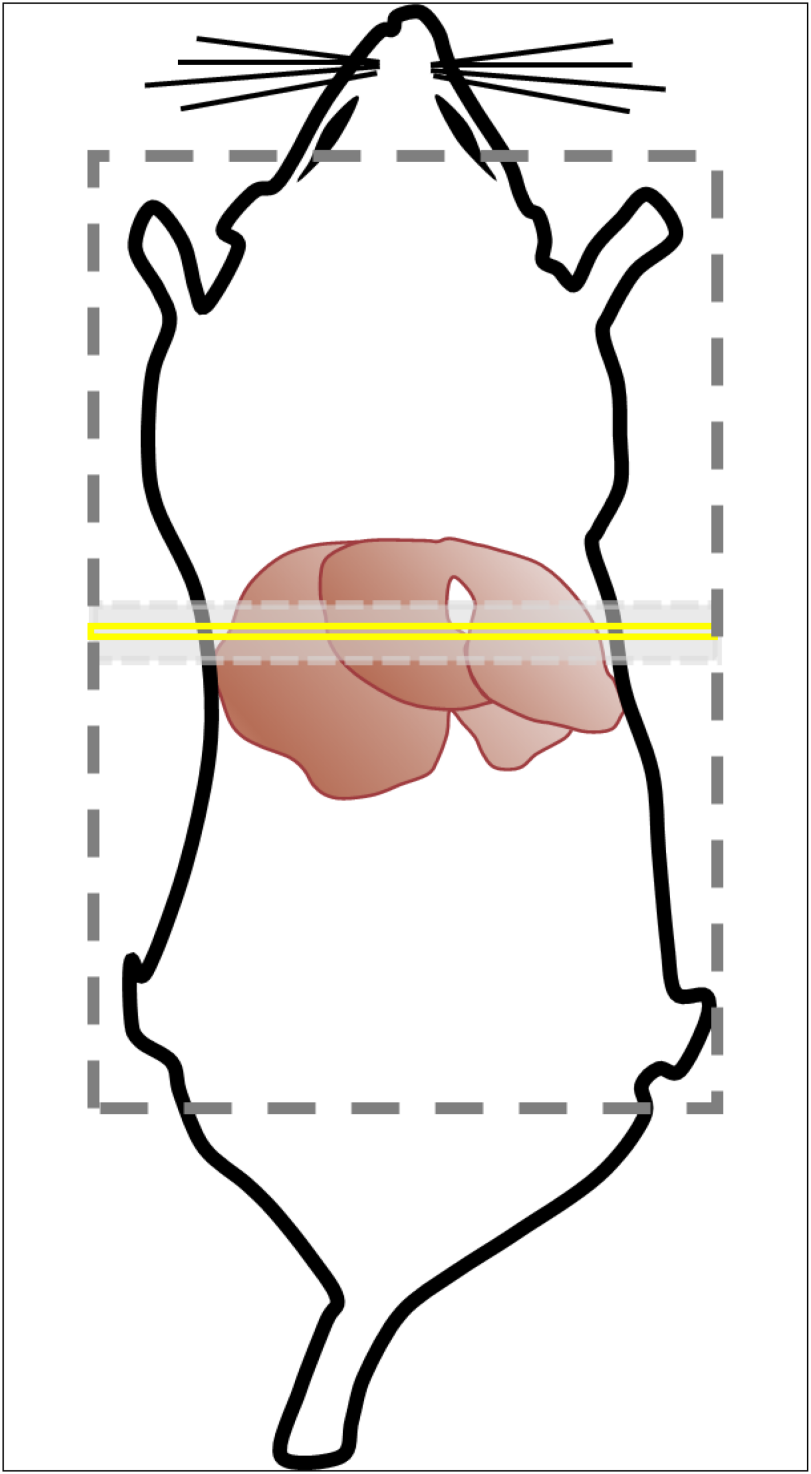
FAIR ASL study planning, demonstrated on a schematic image of the rat and its liver. A 2 mm slice was selected to maximise the overall volume of axially imaged liver parenchyma (yellow box). A 6 mm slice‐selective inversion slab (light grey dashed box) and 200 mm global inversion slab (dark grey dashed box) were centred around the imaging slice. Note that the schematic image and planning slice/slabs shown are not to scale

An axial slice was selected from anatomical imaging that maximised the volume of hepatic parenchyma imaged. A FAIR Look‐Locker ASL sequence was used to measure perfusion using an end‐expiration triggered segmented acquisition with a spoiled gradient‐echo readout.^9^ Data were acquired using a 60 x 60 mm^2^ field of view, 128 x 128 acquisition matrix, 2 mm slice thickness with an echo time (TE) of 1.18 ms, Look‐Locker inversion time (TI_Look‐Locker_) of 110 ms, repetition time (TR_RF_) of 2.3 ms, Look‐Locker flip angle (α_LL_) of 8° and TR_Inversion_ of 13 seconds. Fifty inversion recovery read‐outs were used for each T1 measurement. A 6 mm slice‐selective and 200 mm global inversion slab were used centred on the slice of interest (Figure 2). Four lines of *k*‐space were obtained per segmented acquisition, with a total acquisition time of ~15 minutes per measurement. Because of the large number of TIs, gating was optimised retrospectively using a digital data‐logger (Cambridge Electronic Design, Cambridge, UK), with discarding of any *k*‐space lines acquired during inspiration/expiration.^9^ Selected images were then smoothed using a Gaussian window (σ = 1.6 pixels, final resolution = 753 μm full‐width at half‐maximum), before pixel‐wise T1 measurement.

Postinversion T1 recovery signal at each TI was fitted to Equation 1, to estimate “*M*_0_” (the signal intensity at equilibrium magnetisation), “*α*” (the apparent inversion efficiency) and “*T*1^*^” (the apparent T1), which was then converted to actual T1 using the Look‐Locker correction factor based on small angle approximation (Equation 2)^17^:

(1) $$M_{z}=M_{0}\left(1-\alpha \cdot e^{-\frac{\mathrm{TI}}{T1^{*}}}\right)$$

(2) $$T1=T1^{*}\cdot \left(\alpha -1\right)$$

Flow‐sensitised (slice‐selective) and control (global) T1 maps, assuming constant blood inflow, were then used to generate perfusion (P) maps using^18^:

(3) $$P=\frac{\lambda }{T1_{\text{blood}}}\left(\frac{T1_{\text{global}}}{T1_{\text{slice selective}}}-1\right),$$

where “*λ*” represents the blood‐tissue partition coefficient, reported as 0.95 ml/g (from ^85^Kr gas clearance measurements^19^) and *T*1_*blood*_ was assumed to be 1900 ms from previous work on the same scanner.^20^ Automatic thresholding was used for nonphysiological (>2000 ml/min/100 g) perfusion, with negative perfusion values set to zero.

### 2.5 Liver tissue T1

Parenchymal T1 measurements were derived from control (global, nonflow‐sensitised) T1 maps.

### 2.6 FAIR ASL/liver tissue T1 image analysis

Perfusion and parenchymal liver T1 measurements were obtained from the average of three identically sized circular regions of interest (ROIs) placed on the right, middle and left hepatic parenchyma, avoiding major vascular structures and extra‐hepatic tissue. ROIs were placed via joint consensus between a radiology research fellow (MDC) and imaging scientist (RR), each with more than 4 years of experience of quantitative liver imaging. Researchers were blinded to disease status or inflammatory stress during ROI placement, but ROIs were colocalised to ensure perfusion/T1 was measured at similar baseline/postinflammatory stress sites (as the animal was not physically moved between these studies). Final perfusion and T1 measurements were made by averaging three ROIs. Image postprocessing, fitting and analysis were performed using Matlab (MathWorks, Natick, MA, USA).

### 2.7 Caval subtraction PCMRI

Bulk‐flow measurements were performed as described previously.^15^ Briefly, gradient‐echo anatomical imaging was used for planning PCMRI studies (Table 1). Immediately after FAIR ASL perfusion measurement, cardiac and respiratory‐gated two‐dimensional cine PCMRI studies of the infra‐hepatic supra‐renal inferior vena cava (IVC, *V*_*enc*_ = 33 cm/s) and supra‐hepatic sub‐cardiac IVC (*V*_*enc*_ = 66 cm/s), each with 12‐15 phases through the cardiac cycle (approximate heart rate range 300‐400 beats per minute) were completed within ~10 minutes (Table 1). The voxel width (0.208 mm^2^) was well below one third of the caval vessel diameter (3‐4 mm) to avoid partial volume effect bulk‐flow underestimation.^21^ Manually positioned ROIs were placed on each vessel for each frame of the cardiac cycle using Matlab code developed in‐house. The flow for each frame of the cardiac cycle was measured by summing the product of the voxel area and measured velocity vectors within each ROI. Flow measurements through the cardiac cycle were plotted over time, with the area under the curve used to estimate gross bulk flow (ml/min). TLBF was estimated from the difference between the two caval flow measurements, normalised to explanted liver weight (ml/min/100 g).

### 2.8 Power calculations and statistical analysis

Sample size calculations were based on ACLF studies analysed with mixed‐model two‐way analysis of variance (ANOVA, sham/BDL vs baseline/post‐LPS). Differences in liver blood flow/liver perfusion were used as the endpoint variable. At 90% power and a 5% significance level to detect a change in liver perfusion of at least 20% between sham and BDL rates (informed from clinical ACLF studies^5^), six subjects per group were required. Assuming an attrition rate of 30% (based on prior experience of LPS in BDL rats), the final projected sample size was nine per group. An additional sham‐operated rat from protocol development work was also included (final sham group size, *n* = 10).

Data distribution normality was confirmed using Kolmogorov–Smirnov testing. Agreement between FAIR ASL liver perfusion and caval subtraction PCMRI TLBF was assessed using Bland–Altman analysis by calculating 95% limits‐of‐agreement (LoAs). Calculated coefficients of variation (CoVs) for inter‐subject variation were compared using methods described by Forkman.^22^ Baseline group features, FAIR ASL liver perfusion and tissue T1 were compared using unpaired Student t‐tests, with Welch's correction to account for group differences in standard deviation. Where appropriate, nonparametric Mann–Whitney U‐tests were used for samples that were nonnormal. Paired baseline and post‐LPS measurements of FAIR ASL liver perfusion and tissue T1 in sham and BDL cohorts were evaluated using mixed‐model two‐way ANOVA, citing the *F* (between‐groups degrees of freedom, within‐groups degrees of freedom) statistic with post hoc testing using Bonferroni's correction. Data are expressed as means ± standard errors, with significance at the 5% threshold.

## 3 RESULTS

### 3.1 Subjects

Presurgical weights were similar in both cohorts (250‐300 g), however, 4 weeks after surgery, whole body weight was lower in BDL (401 ± 11 g, *n* = 9) compared with sham‐operated animals (466 ± 7 g, *n* = 10; *P* = .0003), but wet liver mass was higher in BDL (30 ± 2 g) compared with sham‐operated animals (14 ± 1 g; *P* < .0001; Table 2).

**TABLE 2:** Baseline sham‐operated and BDL rat differences

	Sham	BDL	*P*‐value
Cohort features			
Body weight (g)	466 ± 7	401 ± 11	.0003**
Wet liver weight (g)	14 ± 1	30 ± 2	<.0001***
FAIR ASL liver perfusion (ml/min/100 g)	316 ± 24	199 ± 32^†^	.0101*
Liver tissue T1 (ms)	1256 ± 18	1533 ± 50	.0004**

^†^ Mann–Whitney U‐test;

* *P* < .05;

** *P* < .001;

*** *P* < .0001.

### 3.2 Agreement between FAIR ASL liver perfusion and caval subtraction PCMRI

**FIGURE 3:**
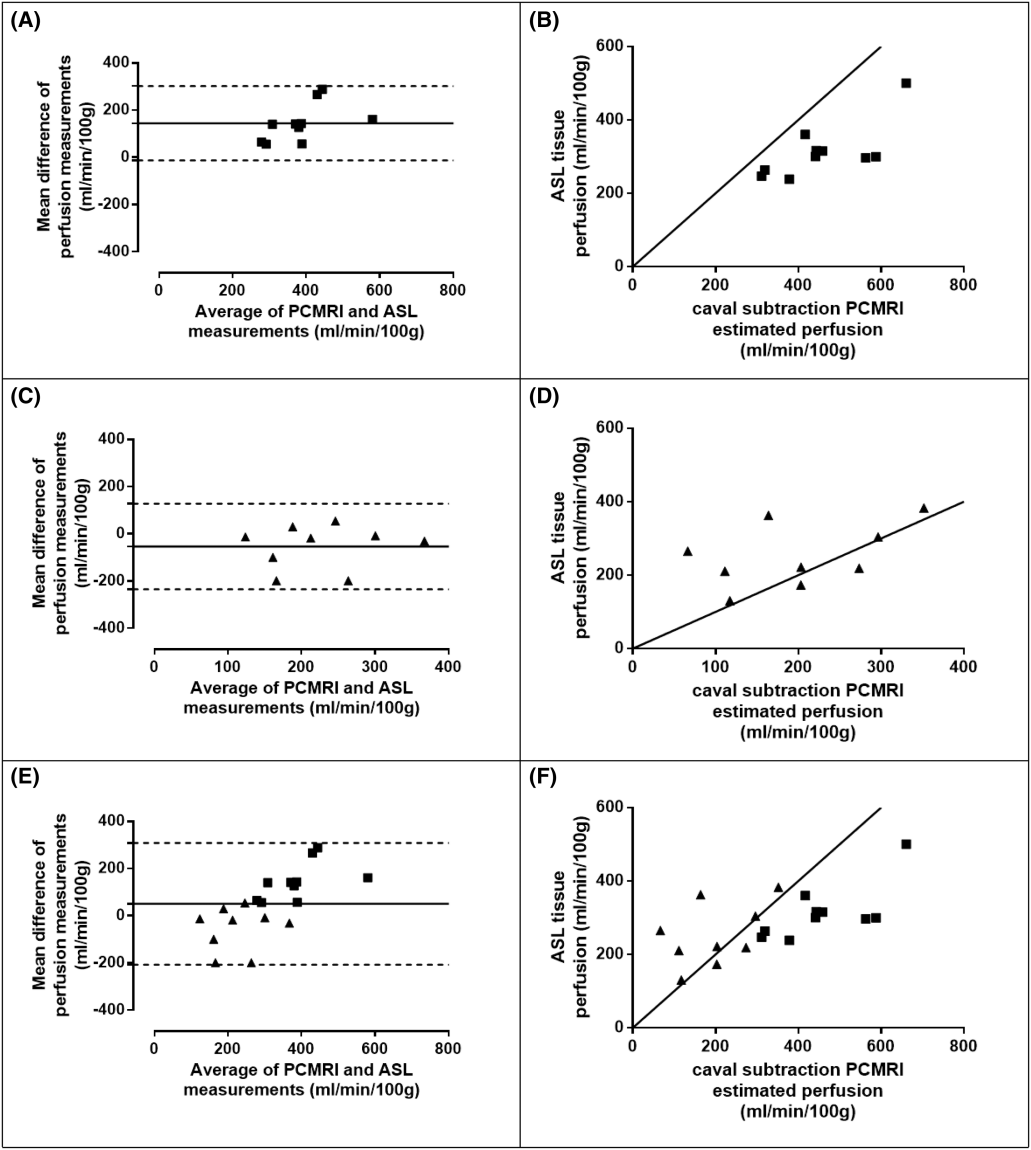
Agreement between FAIR ASL liver perfusion and caval subtraction total liver blood flow for sham‐operated (■) and BDL (▲) rats, using A, C and E, Bland–Altman charts, and B, D and F, scatter plots, with lines of unity shown as solid lines. Summarised statistics are shown in Table 3

The mean difference between FAIR ASL hepatic perfusion (mean 285 ± 19 ml/min/100 g) and caval subtraction PCMRI TLBF (mean 335 ± 39 ml/min/100 g) was 51 ± 30 ml/min/100 g (*P* = .112). The Bland–Altman 95% LoAs for FAIR ASL perfusion vs caval subtraction PCMRI TLBF were ±258 ml/min/100 g (range 130‐500 ml/min/100 g and 66‐661 ml/min/100 g, respectively), with a tendency for FAIR ASL hepatic parenchymal perfusion measurements to be lower than caval subtraction PCMRI (−51 ml/min/100 g) (Figure 3E). Systematic differences were also dependent on animal type (Figure 3 and Table 3). The CoV for FAIR ASL (29.3%) was significantly smaller than for caval subtraction PCMRI (50.1%; F [18, 18] = 0.39; *P* = .0266).

**TABLE 3:** Agreement statistics between FAIR ASL liver perfusion and caval subtraction total liver blood flow for sham‐operated and BDL rats

	Bias (ml/min/100 g)	Bland–Altman 95% Limits‐of‐agreement (ml/min/100 g)	Correlation	
			Coefficient	*P*‐value
Overall	51 ± 30	±258	0.6374*	.0033
Sham	144 ± 25	±157	0.5879^†^	.0806
BDL	−54 ± 31	±181	0.4698	.2019

^†^ Spearman's rho;

* *P* < 0.05).

### 3.3 Baseline FAIR ASL liver perfusion and tissue T1 differences

The results are summarised in Table 2. Baseline sham FAIR ASL liver perfusion was found to be not normally distributed (Kolmogorov–Smirnov distance = 0.2755; *P* = .0302) with inspection of the distribution suggestive of weighting towards the lower end of the range of measured perfusion values (range 238‐500 ml/min/100 g, median 308 ml/min/100 g). Baseline FAIR ASL liver perfusion was significantly lower in BDL (199 ± 32 ml/min/100 g) compared with sham‐operated animals (316 ± 24 ml/min/100 g; Mann–Whitney U‐test, *P* = .01). Conversely, liver tissue T1 was significantly higher in BDL (1533 ± 5 ms) compared with sham‐operated rats (1256 ± 18 ms; *P* = .0004).

### 3.4 Inflammatory stress studies

**FIGURE 4:**
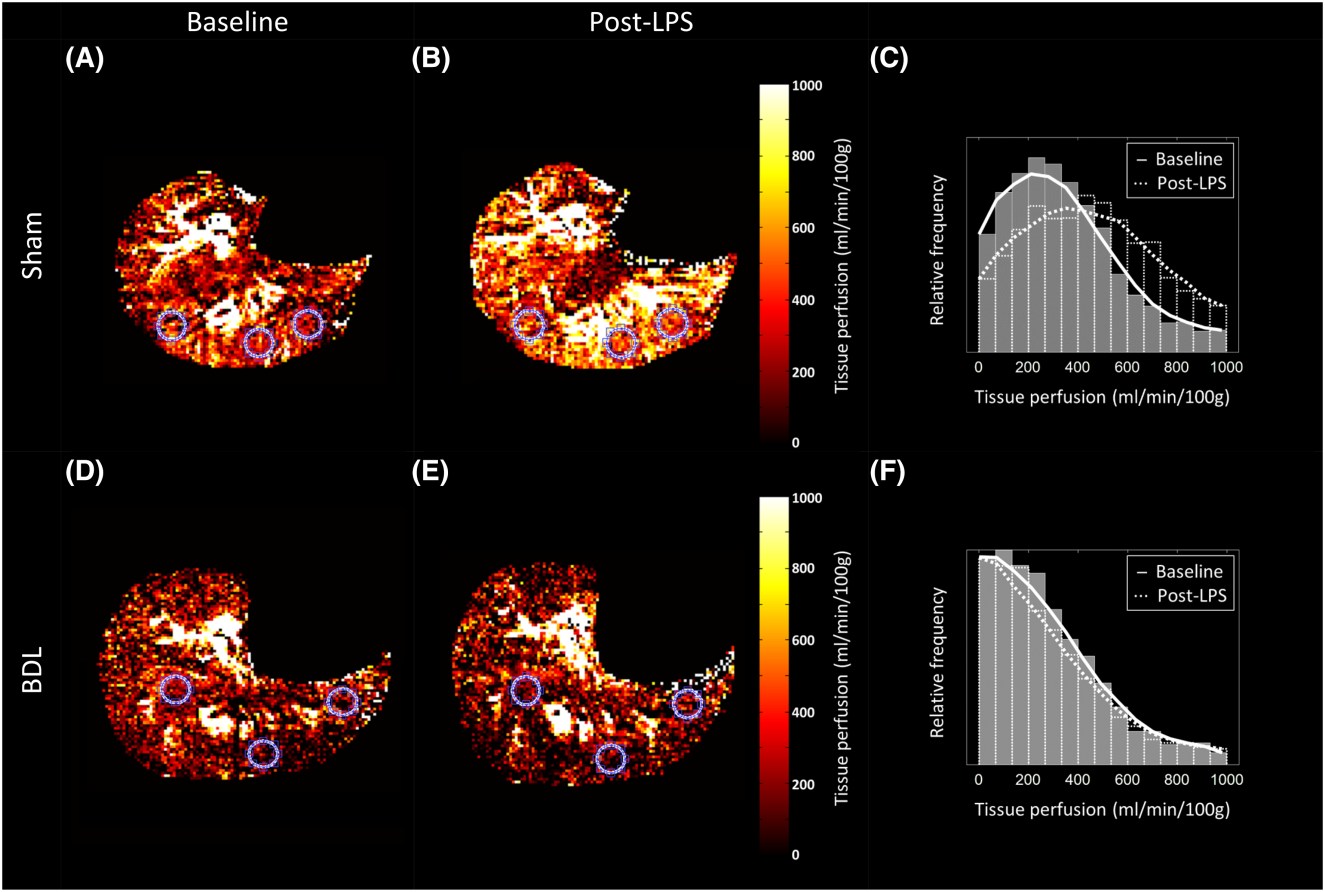
Axial FAIR ASL liver perfusion map examples in A and B, sham‐operated, and D and E, BDL rats at, A and D, baseline, and B and E, post‐LPS. Colocalised circular ROIs used for average parenchymal perfusion measurements are also shown. Baseline (solid grey bars) and post‐LPS (transparent dotted‐outline bars) frequency histograms of the entire imaged slice have been plotted for C, the sham and F, BDL rat perfusion maps shown. Averaged baseline (solid line) and post‐LPS (dotted line) curves have been overlaid to highlight the different perfusion frequency profiles. For the perfusion maps shown, the post‐LPS mode increases for the sham rat, but is similar to baseline in the BDL rat

**FIGURE 5:**
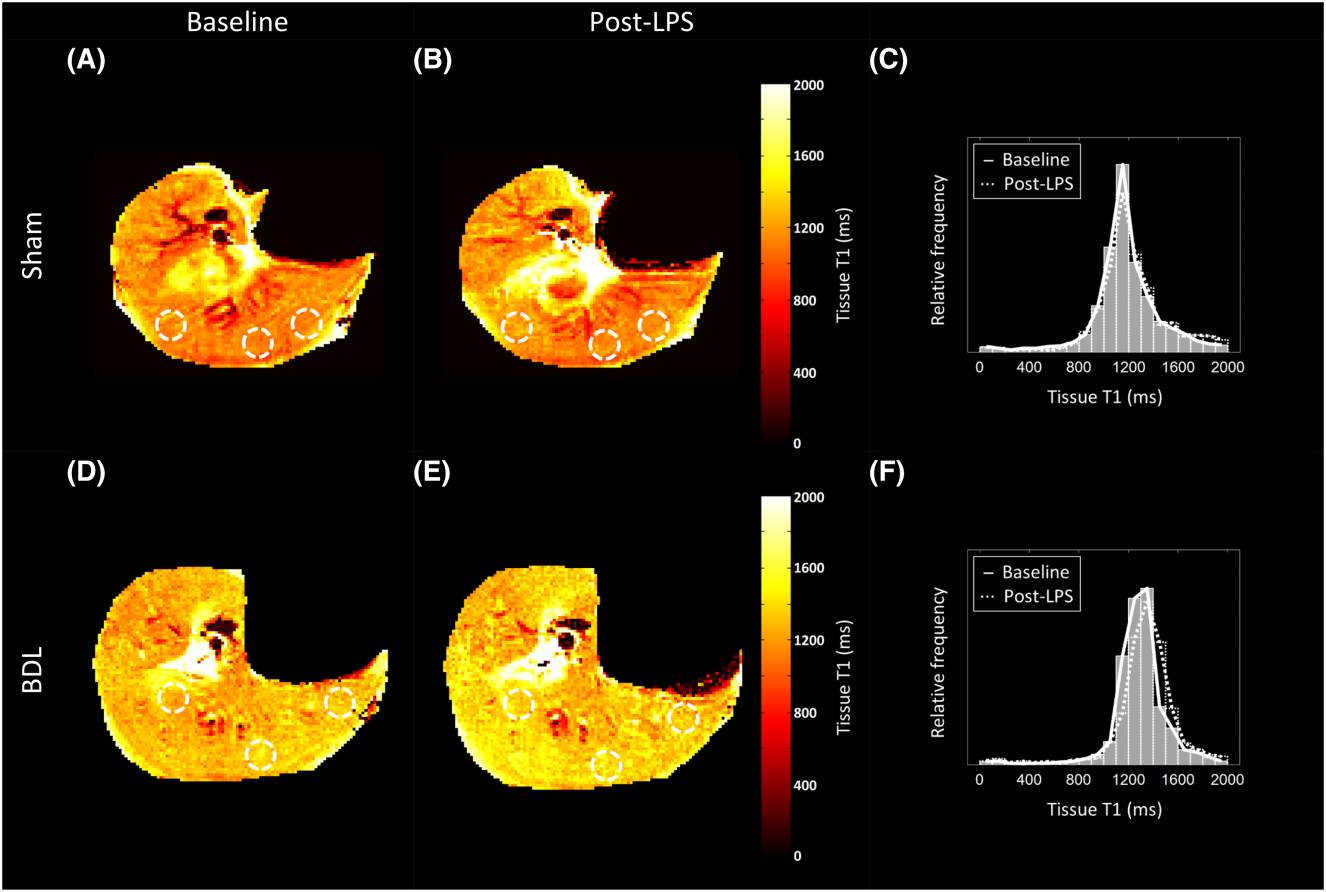
Axial liver T1 map examples from the same A and B, sham‐operated, and D and E, BDL rats, at A and D, baseline, and B and F, post‐LPS shown in Figure 4. Colocalised white circular ROIs used for average parenchymal perfusion measurements are also shown. Baseline (solid grey bars) and post‐LPS (transparent dotted‐outline bars) frequency histograms of the entire imaged slice have been plotted for C, the sham and F, BDL rat T1 maps shown. Averaged baseline (solid line) and post‐LPS (dotted line) curves have been overlaid to highlight the different perfusion frequency profiles. For the T1 maps shown, the mode remains unchanged for both sham and BDL rats post‐LPS

Post‐LPS measurements were obtained on average 28.9 ± 2.5 minutes after completion of the LPS infusion (Figures 4 and 5). The results are summarised in Table 4. Premature demise resulted in completed post‐LPS measurements being collected in seven BDL subjects.

**TABLE 4:** Change in sham and BDL FAIR ASL liver perfusion and tissue T1 in response to LPS

	Sham		BDL		Two‐way ANOVA	
		*P*‐value^†^		*P*‐value^†^	*F* (1,15)	*P*‐value
FAIR ASL liver perfusion (ml/min/100 g)	78 ± 33	.0582	−49 ± 40	.4675	6.248	.0245*
Liver tissue T1 (ms)	0.2 ± 9	>.9999	14 ± 11	.4524	0.9112	.3529

^†^ *P*‐value for post hoc baseline vs post‐LPS;

* *P* < .05;

** *P* < .001;

*** *P* < .0001.

**FIGURE 6:**
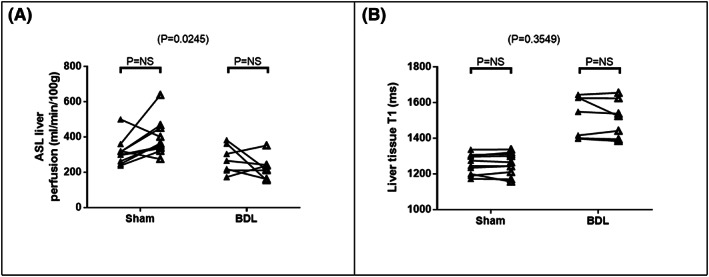
Baseline (▲) and post‐LPS (Δ) changes in A, FAIR ASL liver perfusion, and B, liver tissue T1 in sham‐operated and BDL rats. Two‐way ANOVA *P*‐values are cited above each chart, with post hoc tests cited for each animal type

Statistically significant interactions between animal type and the effects of LPS were demonstrated for FAIR ASL liver perfusion (*F* [1, 15] = 6.248; *P* = .0245) but not for liver tissue T1. Sham rats demonstrated a rise (+78 ± 33 ml/min/100 g) while FAIR ASL liver perfusion fell in BDL rats (−49 ± 40 ml/min/100 g), but post hoc tests were nonsignificant (Figure 6A).

An interaction between animal type and effects of LPS on liver tissue T1 was not demonstrated. No significant change in liver tissue T1 post‐LPS was noted for either sham or BDL animals (Figure 6B).

## 4 DISCUSSION

We have demonstrated that FAIR ASL measurements of hepatic perfusion are feasible in cirrhotic rats and can be used to investigate haemodynamic phenomena in the setting of super‐added inflammation and sepsis. Following bile duct ligation surgery, rats develop a reduction in body weight and increase in liver volume, phenotypically compatible with the evolution of chronic liver disease. We have shown modest agreement with previously invasively validated caval subtraction PCMRI TLBF and that FAIR ASL tends to underestimate hepatic perfusion relative to that suggested by PCMRI, across both animal groups. Looking at data distribution by animal type, the tendency of FAIR ASL to underestimate liver perfusion seems larger in sham animals, where PCMRI measurements are higher. The cause of this trend is unclear: at high field strength this could be due to the effects of inversion pulse radiofrequency bandwidth on labelling efficiency,^23^ but FAIR ASL underestimation in the setting of higher PCMRI bulk flow has been reported previously in clinical studies (on different scanners and with different sequence parameters).^8^ Measured LoAs with PCMRI are wide, but data distribution again suggests these may be smaller if analysed by animal type. The smaller FAIR ASL CoV suggests measurements of hepatic perfusion are less sensitive than when estimated with PCMRI, but are still larger than for previously reported FAIR ASL measurements on the same scanner in mice.^9^ This may reflect some of the limitations of caval subtraction PCMRI TLBF, which suffers from propagation of flow measurement errors from each vessel used in the subtraction.^15^ In this study, invasive reference standards were not used for validation as such experiments would preclude reliable post‐LPS studies and require larger numbers of animals. It is worth noting that caval subtraction PCMRI estimates TLBF, which while inherently different, is (as demonstrated by the presented data) related to tissue perfusion.

We observed reduced liver tissue perfusion and increased T1 at baseline in BDL rats. This is corroborated by clinical FAIR ASL measurements in patients with liver disease, where liver perfusion was reported to be lower in cirrhotic patients than healthy volunteers,^8^ and found to increase in chronic hepatitis C virus‐infected patients following treatment^24^ (ie, suggesting it reflects disease severity and potentially reduced liver inflammation). Several studies have also reported increased liver T1 in patients with liver fibrosis, as demonstrated by our data. T1 maps obtained in this study after the global inversion in FAIR ASL are Look‐Locker T1 maps, which are of comparable accuracy with those obtained by previously reported liver T1 mapping techniques such as modified Look‐Locker and inversion‐recovery methods.^10^, ^25^ Following endotoxin challenge, sham and BDL animals exhibited differing haemodynamic responses, with increased liver perfusion in sham animals (albeit just failing to reach our predetermined level of statistical significance on post hoc testing) but not in BDL animals. Conversely, reductions in bulk hepatic flow measurements in naïve rats following endotoxaemia have been reported previously, but this may reflect the almost seven times higher LPS doses used in that study.^26^ Reductions in invasive indocyanine green (ICG) determined TLBF have been demonstrated previously in patients with ACLF, relative to those with stable cirrhosis.^5^ FAIR ASL liver perfusion measurements in seven patients with decompensated liver disease were also lower than in patients with compensated cirrhosis.^8^ The mechanisms regulating changes in hepatic perfusion induced by acute inflammation in the setting of advanced cirrhosis are complex, incompletely understood and likely multifactorial. The evaluation of liver perfusion in small animals in the context of LPS challenge (emulating ACLF in BDL rats) is demonstrated here for the first time, underscoring the potential use of this method as a tool to improve pathophysiological understanding of ACLF.

Liver tissue T1 was convincingly unchanged for both sham and BDL animals in the setting of LPS challenge. Previously reported data in patients with advanced fibrosis also failed to demonstrate changed liver tissue T1 in the presence of histologically proven inflammation, but did demonstrate inflammation‐related increase in liver T1 for patients with absent/mild fibrosis.^10^ Using the principle that tissue water content is the most significant determinant of T1, it has been proposed that increases in liver T1 observed in patients with chronic liver disease are attributable in part to inflammation‐associated tissue oedema, thereby laying claim to the use of liver T1 as a measure of liver inflammation.^27^ In this study, the demonstration of increased liver T1 in the setting of advanced liver disease, without any change in liver T1 following such a massive inflammatory insult, is an important finding in relation to the use of liver T1 as a biomarker for liver disease, with our data suggesting that changes in liver T1 are more likely driven by fibrosis than by inflammation. Although correlative histological data were not available for our study, acute hepatic tissue inflammation would be expected post‐LPS infusion, as substantiated by studies using comparable LPS doses administered intraperitoneally (ie, with lower bioavailability) and within 1 hour, particularly in the BDL cohort.^12^, ^16^ Arguably, over an extended time period inflammation‐related change in liver T1 could potentially occur, but the absence of any convincing change ~90 minutes after initiating the LPS infusion highlights a potential limitation of liver T1 measurements in patients with liver disease, where the onset of tissue inflammation is of clinical interest.^11^ The relative stability of paired baseline and post‐LPS liver tissue are, however, indicative of the repeatability of liver T1 measurements, despite systemic inflammatory/haemodynamic stress.

Our study does have important limitations. Acquisition of a complete liver FAIR‐ASL/T1 measurement required ~15 minutes, during which time systemic and hepatic haemodynamic factors in the post‐LPS setting, likely fluctuated. Measuring mean arterial pressure would provide a means of monitoring systemic cardiovascular response, but this was not possible because of the need for additional monitoring equipment in the scanner. LPS is also known to cause progressive multi‐organ failure and eventual demise, particularly of BDL rats,^28^ indeed substantial reductions in liver perfusion for two subjects in both the sham and BDL cohorts (Figure 6A) may reflect early/evolving systemic haemodynamic compromise. Two BDL rats failed to complete our protocol, thereby potentially introducing selection bias, as only those animals with sufficient reserve to complete the study were included.

Our experience also highlighted a number of methodological considerations when measuring liver FAIR ASL perfusion/tissue T1. Increased signal‐to‐noise ratio (SNR) at higher field strength permitted ASL measurements without the need for multiple averages, but overall acquisition time was still extended because of the need for retrospective gating to deal with respiratory motion. Measurements were also obtained for a single slice only, when whole‐liver coverage or at least multi‐slice measurements would be more desirable. Perfusion was measured using the Belle et al model,^18^ which was originally developed for cardiac perfusion. Assumed constants such as the blood‐tissue partition coefficient (“λ”, which may vary in liver disease) and blood T1 (which may vary with pyrexia or dilution from intravenous fluid infusion) have the potential to affect overall quantification in both groups differently. FAIR ASL quantification relies on the accuracy of the T1 measurements underpinning the acquisition. Relative baseline/post‐LPS stability is encouraging, and the comparison of global/slice‐selective T1 maps would have reduced errors introduced by B1 inhomogeneities, particularly at high field strength.^29^ Finally, care was taken to minimize fluctuations in anaesthetic dosage: large variations have the potential to exert systemic haemodynamic effects, although previous preclinical studies have shown that hepatic perfusion is relatively robust to variations in isoflurane dosage.^9^, ^30^

This study has identified several areas for future work. Although repeatability of FAIR ASL liver perfusion measurements in mice has been previously reported,^9^ the modest agreement demonstrated between FAIR ASL perfusion and PCMRI TLBF in rats underscores the importance of repeatability/reproducibility studies as a means of understanding measurement confidence and their potential use going forward. FAIR ASL is a pulsed ASL method appropriate for the measurement of global tissue perfusion, but is unable to separate HA and PV contributions. Pseudo‐continuous ASL methods have the potential to address this, which would be meaningful in chronic liver disease, where changes in the relative HA/PV contributions may have prognostic implications.^1^ We have demonstrated differences in tissue T1, but the compositional changes in liver tissue that underpin this are still poorly understood. Liver inflammation (in this study), and qualitative fat and iron content have been explored previously,^10^ but true quantitative studies of these and compositional changes such as collagen deposition and neovascularisation are needed, if the value of liver tissue T1 in clinical practice is to be understood. Finally, our studies have demonstrated the potential of noninvasive MRI measurements of hepatic tissue perfusion in following inflammatory stress in a small animal model of chronic liver disease. Studies evaluating the effects of systemic mediators and new vasoactive drugs could be used to develop new and much needed therapies for acute and chronic inflammation in the setting of liver disease, such as ACLF or NASH.

In summary, we have demonstrated that FAIR ASL measurements of hepatic perfusion can be used to investigate hepatic haemodynamic phenomena in a small animal model of chronic liver disease. FAIR ASL hepatic perfusion measurements agree modestly with validated caval subtraction PCMRI measurements of TLBF, and tend to underestimate larger measurements. At baseline we have noninvasively shown reduced liver perfusion in BDL rats, and increased liver T1 suggestive of hepatic fibrosis. We have also demonstrated an altered hepatic haemodynamic response in response to inflammatory stress, and with stability of liver T1 measurements in sham and BDL rats, thereby questioning the use of liver tissue T1 as a measure of hepatic inflammation. Finally, our study highlights the role of small animal hepatic FAIR ASL as a potentially useful noninvasive tool for the assessment of liver perfusion and therapeutic response to new vasoactive therapies for patients with chronic liver disease.

## FUNDING INFORMATION

This study was funded by: Wellcome Trust Clinical Research Training Fellowship (grant WT092186); Wellcome Trust Senior Research Fellowship (grant WT100247MA); MRC Capacity Building Studentship; British Heart Foundation; King's College London and UCL Comprehensive Cancer Imaging Centre CR‐UK & EPSRC; and National Institute of Health Research University College London Hospitals Biomedical Research Centre.
